# Influenza A virus NS1 gene mutations F103L and M106I increase replication and virulence

**DOI:** 10.1186/1743-422X-8-13

**Published:** 2011-01-12

**Authors:** Samar K Dankar, Shuai Wang, Jihui Ping, Nicole E Forbes, Liya Keleta, Yishan Li, Earl G Brown

**Affiliations:** 1 Department of Biochemistry, Microbiology and Immunology, Faculty of Medicine, University of Ottawa 451 Smyth Rd., Ottawa, Ontario, K1H 8M5, Canada; 2 Emerging Pathogens Research Centre, Faculty of Medicine, University of Ottawa, 451 Smyth Rd., Ottawa, Ontario, K1H 8M5, Canada; 3 Laboratory of Animal Infectious Diseases, College of Veterinary Medicine, China Agricultural University, Beijing, 100094 PR China

## Abstract

**Background:**

To understand the evolutionary steps required for a virus to become virulent in a new host, a human influenza A virus (IAV), A/Hong Kong/1/68(H3N2) (HK-wt), was adapted to increased virulence in the mouse. Among eleven mutations selected in the NS1 gene, two mutations F103L and M106I had been previously detected in the highly virulent human H5N1 isolate, A/HK/156/97, suggesting a role for these mutations in virulence in mice and humans.

**Results:**

To determine the selective advantage of these mutations, reverse genetics was used to rescue viruses containing each of the NS1 mouse adapted mutations into viruses possessing the HK-wt NS1 gene on the A/PR/8/34 genetic backbone. Both F103L and M106I NS1 mutations significantly enhanced growth *in vitro *(mouse and canine cells) and *in vivo *(BALB/c mouse lungs) as well as enhanced virulence in the mouse. Only the M106I NS1 mutation enhanced growth in human cells. Furthermore, these NS1 mutations enhanced early viral protein synthesis in MDCK cells and showed an increased ability to replicate in mouse interferon β (IFN-β) pre-treated mouse cells relative to rPR8-HK-NS-wt NS1. The double mutant, rPR8-HK-NS-F103L + M106I, demonstrated growth attenuation late in infection due to increased IFN-β induction in mouse cells. We then generated a rPR8 virus possessing the A/HK/156/97 NS gene that possesses 103L + 106I, and then rescued the L103F + I106M mutant. The 103L + 106I mutations increased virulence by >10 fold in BALB/c mice. We also inserted the avian A/Ck/Beijing/1/95 NS1 gene (the source lineage of the A/HK/156/97 NS1 gene) that possesses 103L + 106I, onto the A/WSN/33 backbone and then generated the L103F + I106M mutant. None of the H5N1 and H9N2 NS containing viruses resulted in increased IFN-β induction. The rWSN-A/Ck/Beijing/1/95-NS1 gene possessing 103L and 106I demonstrated 100 fold enhanced growth and >10 fold enhanced virulence that was associated with increased tropism for lung alveolar and bronchiolar tissues relative to the corresponding L103F and I106M mutant.

**Conclusions:**

The F103L and M106I NS1 mutations were adaptive genetic determinants of growth and virulence in both human and avian NS1 genes in the mouse model.

## Introduction

IAV have now caused 4 pandemics in the past century, the most lethal being the 1918 Spanish Flu pandemic, where global mortality exceeded 20 million [1]. In addition, several new viral subtypes with presumed pandemic potential have arisen including virulent avian strains of H7N7 [2], H9N2 [3] and H5N1 [4] that have demonstrated an increased capability to infect, replicate and cause severe disease in humans. There is thus a need to identify genetic mutations that control host range and virulence so that viruses with the potential to cause virulent pandemics in humans can be identified and monitored.

Increased virulence of avian HPAI H5 and H7 IAVs require the presence of a multi-basic amino acid HA cleavage site [5], however this feature is not sufficient to confer high virulence and further analysis indicates that virulence is polygenic and requires additional mutant genes [6]. Key mutations in the PB2 gene increased pathogenicity and viral transmission such as E627K and D701N [7] and mutation sites in H3 HA1 and HA2 subunits, G218W and T156N respectively, have been shown to affect both growth and virulence in the mouse [8]. Further analysis of A/HK/1/68 (H3N2) mouse adapted mutations of the HKMA variant demonstrate that all mutated genome segments enhanced disease severity including the NS1 V23A mutation that increased virulence by 10^0.8 ^fold [9]. Here, we extend the experimental mouse model to identify two adaptive mutations in the NS1 gene, F103L and M106I that have previously been observed in fatal human infections with A/HK/156/97 (H5N1) [10].

NS1 is a multifunctional protein that binds both ssRNA and dsRNA [11,12] and interacts with a number of host cellular proteins [13,14]. NS1 functions as an IFN antagonist to inhibit IFN production and signaling (reviewed in [13]). NS1 blocks the recognition of dsRNA by the cytoplasmic pathogen recognition receptor RIG-I (retinoic inducible gene 1) [15] and therefore limits the activation of IFN transcription [16,17]. NS1 also acts post- transcriptionaly, to inhibit the 3'-end processing of host mRNA including IFN mRNA by binding to CPSF30 (cleavage and polyadenylation specificity factor 30) [18] and PABPNI (poly-A-binding protein nuclear I) [19]. NS1 also directly binds the IFN effectors PKR and 2'-5' OAS (oligo adenylate synthetase) to counteract inhibition of viral protein synthesis [20] and viral RNA degradation respectively [21]. NS1 is also involved in enhancing viral protein synthesis by interacting with the viral mRNA [22], the translation initiation factors eIF4GI (eukaryotic initiation factor 4GI) [23], and PABPI (poly-A-binding protein 1) [24]. NS1 can also limit the early induction of apoptosis by interacting with Pi3K and inducing Akt phosphorylation [24,25]. Although some of these functions attributed to NS1 can vary among strains, these mechanisms (and presumably their modulation) allow NS1 to escape the innate immune response and increase viral replication and growth [13,26].

With respect to the role of the NS1 protein in virulence, previous studies have identified single mutations in the NS1 gene (S42P, D92E and V149A) as well as multiple mutations in the PDZ ligand domain that increased viral pathogenicity [27-30]. Furthermore studies of virulence of pathogenic avian A/HK/156/97-like H5N1 showed that the NS1 gene mediated increased virulence in the mouse model [31] and swine models [27]. In contrast, other studies have identified F103L and M106I mutations in this H5N1 NS1 gene that result in a loss of the ability to bind the cleavage and polyadenylation specificity factor and inhibit IFN induction when transferred into A/Udorn/1/1972 (H3N2) virus [32]. Here we show that the F103L and M106I NS1 gene mutations are adaptive and control IAV replication and virulence.

## Materials and methods

### Cells and viruses

Madin-Darby canine kidney (MDCK) cells, human embryonic kidney cells (293T), mouse kidney epithelial cells (M1), and human lung epithelial cells (A549) were maintained in complete minimum essential medium or Dulbecco's Modified Eagle Medium (Gibco, Carlsbad, USA) supplemented with L-glutamine (2 mM), penicillin (100 U/ml), streptomycin (100 μg/ml) and fetal bovine serum (to 10%).

The prototype A/HK/1/68 (H3N2) (HK-wt) (obtained from the Laboratory center for Disease Control, Health Canada) having an LD_50 _of >10^7.7 ^pfu/ml in mice was used to generate virulent mouse-adapted strains [8,33,34].

### Cloning of the viral NS genes in bidirectional reverse genetics plasmids

The NS cDNA of A/HK/1/68 wt was inserted into pLLB vector by ligation independent cloning [35] and PCR directed mutagenesis was used to introduce point mutations (F103L, M106I, and F103L + M106I) into the HK-wt NS gene. To improve the efficiency of ligation independent cloning, the vector and insert DNA were treated with T4 DNA polymerase before transformation of E. coli as described [36].

The NS genes of A/Chicken/Beijing/1/95-H9N2 (Ck/Bj/95) (obtained from China Agricultural University) and A/Hong Kong/156/97-H5N1 (A/HK/156/97) (Genbank AF036360) (synthesized by Biomatic Corporation, Cambridge, Ontario) were cloned into the pHH21 and pLLB plasmids respectively by homologous recombination as described [37]. The NS1 amino acids 103L and 106I sites of both Ck/Bj/95 and A/HK/156/97 were mutated to 103F and 106M by PCR directed mutagenesis. The remaining 7 gene segments of A/PR/8/34 and A/WSN/33 were obtained from R. Webby (St. Judes Children's Research Hospital, Memphis) and Y. Kawaoka (University of Madison Wisconsin) respectively.

### Reverse genetics

Each of the NS plasmid constructs along with the 7 plasmids encoding the viral structural genes of A/PR/8/34 (0.65 μg/plasmid) were mixed with lipofectamine 2000 (Invitrogen, Carlsband, USA) according to the manufacturer's instructions and incubated for 30 min at RT. The plasmids and the transfection reagent were added into a monolayer of 293T cells, 16 h post transfection, the transfection mix was replaced by opti-mem (Invitrogen, Carlsbad, USA) supplemented with TPCK trypsin (1 μg/ml) (Thermo Fisher Scientific, Ottawa). Forty-eight hours post-transfection the cell supernatant was collected and inoculated in 10-day old embryonated specific pathogen free embryonated eggs (Canadian Food Inspection Agency, Ottawa) for virus propagation at 37°C for 48 hours. The allantoic fluid was collected and viral yield was assessed by plaque assay. The NS gene sequence of the rescued viruses was verified by sequence analysis. The H9N2-NS plasmid was rescued onto the A/WSN/33 backbone using the 12 plasmid system as described [38].

### Growth of the recombinant viruses *in vitro*

Monolayers of MDCK and A549 cells were washed twice with 1 × PBS and infected with each of the four rPR8 viruses at an MOI of 0.001 in presence of TPCK trypsin (1 μg/ml) in triplicate. The supernatant was collected at 12, 24, 48 and 72 hours post infection (hpi) and viral titer was assessed by plaque assay in duplicate for each sample.

### Plaque Assay

Virus samples were serially diluted in PBS. Six well plates of confluent monolayers of MDCK cells were washed twice with PBS and then infected with 100 μl of the different virus dilutions in duplicate for each dilution. The plates were incubated at 37°C for 30 min to allow virus adsorption. Following adsorption, the cells were overlayed with 0.65% agarose (Invitrogen, Carlsbad, USA), in complete MEM supplemented with TPCK trypsin (1 ug/ml). The plates were incubated at 37°C and 3 days post incubation plaques were fixed with Carnoy's fixative (three parts acetic acid to one part methanol v/v).

### Growth of the recombinant viruses *in vivo*

Groups of eleven BALB/c mice (4-to-6-week-old females from Charles River Laboratories, Montreal, Quebec, Canada) were infected intranasally under halothane anesthesia with 5 × 10^3 ^pfu of each of the rPR8 viruses in a volume of 50 μl [34]. Mice were sacrificed at days 1, 2, 3, 5 and 7 post infection. Lungs from two mice were collected for each of the days except for day 2 where three mice were sacrificed. Organs were weighed, diluted 1:4 in PBS and homogenized by sonication for quantification by plaque assay. The same protocol was applied for the rWSN viruses. However, two mice were collected at each of 1, 3, 5 and 7 days post infection (dpi).

### Mouse Survival Assay

Groups of 5 BALB/c mice were infected intranasally with 5 × 10^6 ^pfu of rPR8-HK-NS (NS-WT and variants) and 10^6 ^pfu for the recombinant rWSN viruses. Groups of 3 mice were infected intranasally with 10^4 ^pfu of the rPR8-H5N1-NS viruses. Mortality was monitored for 14 dpi. Body weight was recorded daily and the LD_50 _was calculated by the Karber-Spearman method [39].

### Ethics Statement

The animal studies were carried out in compliance with the guidelines of the Canadian Council on Animal Care (CCAC) as outlined in the Care and Use of Experimental Animals, Vol.1, 2nd Edn. (1993), which are internationally recognized as "best-practices" by the International Council for Laboratory Animal Science (ICLAS). The animal study protocol was approved by the University of Ottawa Animal Care Committee (Protocol Number: BMI-85). Efforts were made to minimize suffering and mice were euthanized at humane end-points when infections resulted in greater than 25% body weight loss plus respiratory distress.

### Immunofluorescence staining of BALB/c lungs

BALB/c mice were infected intranasally with 10^5 ^PFU of each of the recombinant viruses and lungs were collected two dpi. Lungs were inflated and fixed with 3.7% formaldehyde as described earlier [40]. Lung sections were stained as described previously [8]. Rabbit anti-PR8 and anti-WSN primary antibodies that had been pre-adsorbed with mouse lung extract were used for immunohistochemistry. Images were taken at 20 × magnification using an Olympus BX50 microscope and processed in a parallel manner using Photoshop 7.0.

### Rate of viral protein synthesis

Monolayers of MDCK were infected with the different rPR8 viruses at MOI = 3. Two, 4, 6 and 8 hpi, the cells were pulse-labeled with 80 μCi/ml ^35^S cysteine and methionine for one hour in cysteine and methionine free media (Gibco, Carlsbad, USA). Post labeling, the cell lysates were collected with 1 × sodium dodecyl sulfate (SDS) sample buffer. The rate of viral protein synthesis was then assessed by SDS page gel electrophoresis and autoradiography as previously described [41].

### Interferon-β ELISA Assay

Monolayers of M1 cells were infected with each of the rPR8 and rWSN viruses at an MOI of 2. Twenty-four hours post infection the cell supernatants were collected and tested for IFN-β production. Infection of M1 cells with the VSV Indiana Δ 51 mutant that has a defect in the ability to block IFN induction was performed as a positive control using identical conditions [42]. Mouse IFN-β was titrated relative to mouse IFN-β standards by commercial ELISA as described by the manufacturer, PBL Biomedical Laboratories (New Jersey, USA).

### Interferon sensitivity

Monolayers of M1 cells were treated with 200 U/ml of murine IFN-β (Sigma, St. Louis, USA) for 24 hours in triplicate assays. Twenty-four hours post treatment, the cells were washed twice with 1 × PBS prior to infection with the rPR8 viruses at MOI = 1 (1.5 × 10^6 ^PFU per 35 mm dish) for 30 min at 37°C. Following infections, the cells were washed twice with 1 × PBS, and serum free media was applied with TPCK trypsin (1 μg/ml). The supernatant was then collected at 0, 8, 24 and 48 hpi and the viral titer was assessed by plaque assay (in duplicates for each individual infection). The same experiment was done in parallel but without prior IFN-β treatment.

### Statistical analysis

**Figure 1:**
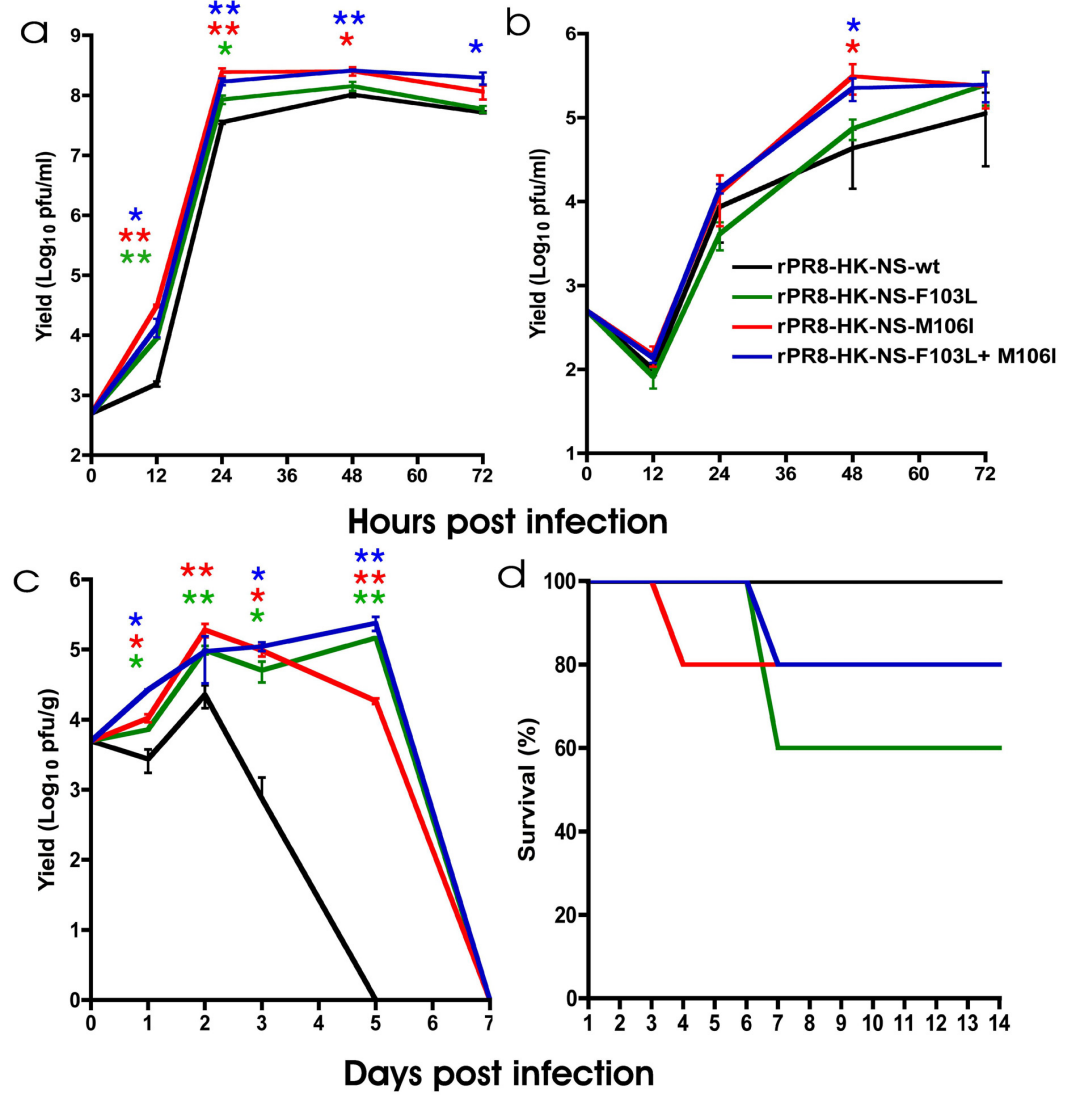
**Increased virulence and viral growth of NS1 mutants in *in vitro *and *in vivo *systems**. A. Growth of rPR8 mutant viruses in MDCK cells. B. Growth of rPR8 mutant viruses in A549 human epithelial cells. Monolayers of MDCK or A549 cells were infected with each of the recombinant viruses at MOI = 0.001 in presence of trypsin. Supernatants were collected at 12, 24, 48 and 72 hpi and viral titer was assessed by plaque assay. Values are shown as averages +/- standard deviation. C. Growth of rPR8 mutant viruses in BALB/c lungs. Groups of 11 BALB/c mice were infected intranasally with 5 × 10^3 ^PFU. Lungs were collected at days 1, 2, 3, 5 and 7 post infection. Organs were sonicated on ice and viral titer was assessed by plaque assay. Values are shown as averages +/- standard deviation at day 3 pi and +/- standard error for the rest of the time points. D. Virulence of rPR8 mutant viruses in BALB/c. Groups of 5 BALB/c were infected intranasally with 5 × 10^6 ^pfu with the rPR8 viruses. Survival was monitored for 14 dpi.

Statistical significance was measured using the student t-test (Numbers, 09 v.2.0.4) using the parameters of equal variance and two-tailed significance (unless otherwise indicated) with a probability of ≤ 0.05 considered as statistically significant. Values were calculated as means +/- standard deviation for sample size > 2 and +/- standard error for sample sizes of 2 (for some of the values in Figures 1c as well as 6b).

## Results

### Sequence Analysis of the mouse adapted strains

In order to identify nonsynonymous NS1 gene mutations that control virulence we generated virulent mouse adapted variants of A/HK/1/68 and compared their NS1 sequence changes to those found in the virulent H5N1 variant A/HK/156/97. Sequence analysis of the NS1 genes of 42 mouse-adapted clones identified two groups of variants MA20-B, -C and -D [33,34] as well as MA-51, -52 and -53 [8,33] (Table 1) that possessed the F103L and M106I mutations respectively and thus were convergent with highly pathogenic H5N1 IAV isolated from humans in Hong Kong in 1997 (A/Hong Kong/156/97-like viruses) [10]. This indicated that these mutations were under positive selection and were therefore functionally important such that they may be involved in increasing replicative fitness and virulence of the mouse adapted variants, MA20c and MA51, with increase in LD_50 _values of 10^2.5 ^and 10^5.5 ^respectively (relative to the HK-wt parent virus, LD_50 _>10^7.7 ^pfu). Because the F103L and M106I mutations have been associated with increased IFN-β induction in reassortants of the A/Hong Kong/156/97(H5N1) NS1 gene on the A/Udorn/1/1972 (H3N2) backbone [32] we measured the IFN-β induction by the parental HK-wt and the MA20c (F103L) and MA51 (M106I) mutant viruses in mouse M1 cells. Using an MOI of 2, the MA20c and MA51 viruses induced low levels of IFN-β after 24 h of incubation that were not significantly different from HK-wt (12 +/- 8 pg/ml).

**Table 1:** NS1 mutations in MA variant clones of A/HK/1/68 (HK-wt) H3N2.

Virus	NS1 Amino Acid positions									
	
	2	23	98	103	106	124	125	180	226	227
HK-wt	D	V	L	F	M	M	D	V	V	R

HKMA20 (MA20)	-	A	-	-	-	-	-	-	-	-

HKMA20a (MA20A)	-	A	-	-	-	-	-	-	-	-

HKMA20b (MA20B)	-	-	-	L	-	-	-	-	-	-

HKMA20c (MA20C)	-	-	-	L	-	-	-	-	-	-

HKMA20d (MA20d)	-	-	-	L	-	-	-	-	-	-

HK4MA21-1 (MA41)	-	-	-	-	V	-	-	-	-	-

HK4MA21-2 (MA42)	-	-	-	-	V	-	-	-	-	-

HK4MA21-3 (MA43)	-	-	-	-	V	I	-	-	-	-

HK5MA21-1 (MA51)	-	-	-	-	I	-	-	-	-	-

HK5MA21-2 (MA52)	-	-	S	-	I	-	-	-	-	-

HK5MA21-3 (MA53)	-	-	S	-	I	-	-	-	-	-

HK6MA21-2 (MA62)	-	-	-	-	-	-	-	A	-	-

HK6MA21-3 (MA63)	-	-	-	-	-	-	-	A	-	-

HK9MA21-3 (MA93)	N	-	-	-	-	-	-	-	-	-

HK10MA21-2 (MA102)	-	-	-	-	-	-	G	-	-	-

HK10MA21-3 (MA103)	-	-	-	-	-	-	-	-	I	-

HK11MA21-1 (MA111)	-	-	-	-	-	-	G	-	-	-

HK11MA21-2 (MA112)	-	-	-	-	-	-	-	-	-	K

### Growth of rPR8 NS1 mutant viruses *in vitro*

To test the role of F103L and M106I mutations on growth, we first generated viruses possessing the HK-wt (106F + 106M), or F103L, M106I and F103L + M106I mutations on the A/PR/8/34 backbone. To compare the growth kinetics of viruses that differed due to specific change in their NS1 genes, rPR8-HK-NS-wt, rPR8-HK-NS F103L, rPR8-HK-NS-M106I, and rPR8-HK-NS-F103L + M106I were used to infect MDCK and A549 epithelial cells, in triplicate, at a low MOI (0.001) to initiate multicycle replication. Supernatants were collected at 12, 24, 48 and 72 hpi and were assayed for yield of infectious progeny virus as measured by plaque assay. All the mutants had a significantly increased ability to grow in MDCK cells compared to rPR8-HK-NS-wt with the rPR8-HK-NS-M106I and rPR8-HK-NS-F103L + M106I mutants growing to higher titers than the rPR8-HK-NS-F103L mutant (Figure 1a). Growth was particularly enhanced at early times (12 and 24 hpi) (P < 0.05) in the three mutant viruses compared to rPR8-HK-NS-wt, where the F103L mutation enhanced yields by 6 and 2 fold respectively; relative to the M106I containing, single and double, mutants that showed 20 and 9, as well as 7 and 5 fold enhanced yields for these respective viruses at 12 and 24 hpi. However, at the 48 hpi time point, only rPR8-HK-NS-M106I, and rPR8-HK-NS-F103L+M106I viruses grew significantly better than rPR8-HK-NS-wt (P < 0.05) and at 72 hpi, the double mutant was the only virus that grew to a significantly higher titer than the rPR8-HK-NS-wt virus (P < 0.05) (Figure 1a).

In contrast, growth of the different mutants in A549 cells was comparable to rPR8-HKNS-wt at early time points (i.e. 12 and 24 hpi), whereas at later time points (48 and 72 hpi) there was a trend to increased yield for the NS1 mutants where the rPR8-HK-NS-M106I and rPR8-HK-NS-F103L + M106I mutants demonstrate statistically significant enhanced growth compared to rPR8-HK-NS-wt at 48 hpi (P < 0.05) (Figure 1b). Therefore, the NS1 mutations increased viral replication and growth *in vitro *compared to the rPR8-HK-NS-wt in MDCK and, in the case of rPR8-HK-NS-M106I and rPR8-HK-NS-F103L + M106I mutants, in A549 cells and thus demonstrate their adaptive nature in both human and canine cells.

### Growth of rPR8 mutant viruses *in vivo*

The three mutant rPR8 viruses along with the rPR8-HK-NS-wt were used to infect groups of 11 BALB/c mice with 5 × 10^3 ^pfu each via the intranasal route, and their lung growth properties in lung tissue were compared. rPR8-HK-NS-wt virus reached a peak titer of 3.7 × 10^4 ^pfu/g in mouse lungs at 2 dpi and was cleared from the lungs by 5 dpi (Figure 1c). All the NS1 adaptive mutations significantly increased replication in mouse lung compared to rPR8-HK-NS-wt at 1 dpi (P < 0.05) and for each of the single mutants 2 days after infection (P < 0.01). Thereafter, viral growth was increasingly enhanced (100 fold at 3 dpi (P < 0.05), and 5 logs by 5 dpi (P < 0.01)) (Figure 1c). Thus all the NS1 mutants delayed viral clearance by 2 days in mouse lungs compared to rPR8-HK-NS-wt. In addition, rPR8-HK-NS-F103L and the double mutant rPR8-HK-NS-F103L + M106I demonstrated enhanced viral growth relative to the rPR8-HK-NS-M106I mutant at 5 dpi (Figure 1c). This indicated that both of the mouse adaptive mutations, F103L and M106I, were associated with increased replication in mouse lungs such that they grew faster and to a higher yield as well as persisted longer in the mouse lung compared to rPR8-HK-NS-wt. The level of IFN-β was undetectable by ELISA in these infected mouse lung extracts. No virus was detected in the liver of any of the mice indicating that the viruses are incapable of growing systemically in BALB/c mice following infection at 5 × 10^3 ^pfu (data not shown).

### Virulence of the rPR8 mutant viruses in BALB/c mice

To compare the virulence of the different recombinant viruses, groups of 5 BALB/c mice were infected intranasally with 5 × 10^6 ^pfu of the different recombinants and mortality was monitored for 14 days pi. Although the LD_50 _of the rPR8 virus was 10^3.5 ^pfu (data not shown), introduction of the HK-wt NS1 gene onto this backbone resulted in rPR8-HK-NS-wt that was avirulent in mice and did not result in mortality (LD_50_>10^7 ^pfu) indicating an important role for PR8 NS1 in virulence (Figure 1d). Similar infection of mice with 5 × 10^6 ^pfu of each of the three mutants resulted in mortality within each group (Figure 1d). A single mutation (F103L) introduced to the HK-wt NS gene resulted in 40% mortality in BALB/c, while the infection with the viruses possessing M106I and F103L + M106I mutations resulted in a 20% mortality rate by 4 and 7 dpi respectively. Consistent with their increased growth in mouse lungs, the NS1 adaptive mutations increased virulence *in vivo *as observed by mortality when compared to the rPR8-HK-NS-wt.

### 103L and 106I increase tropism in BALB/c lungs

**Figure 2:**
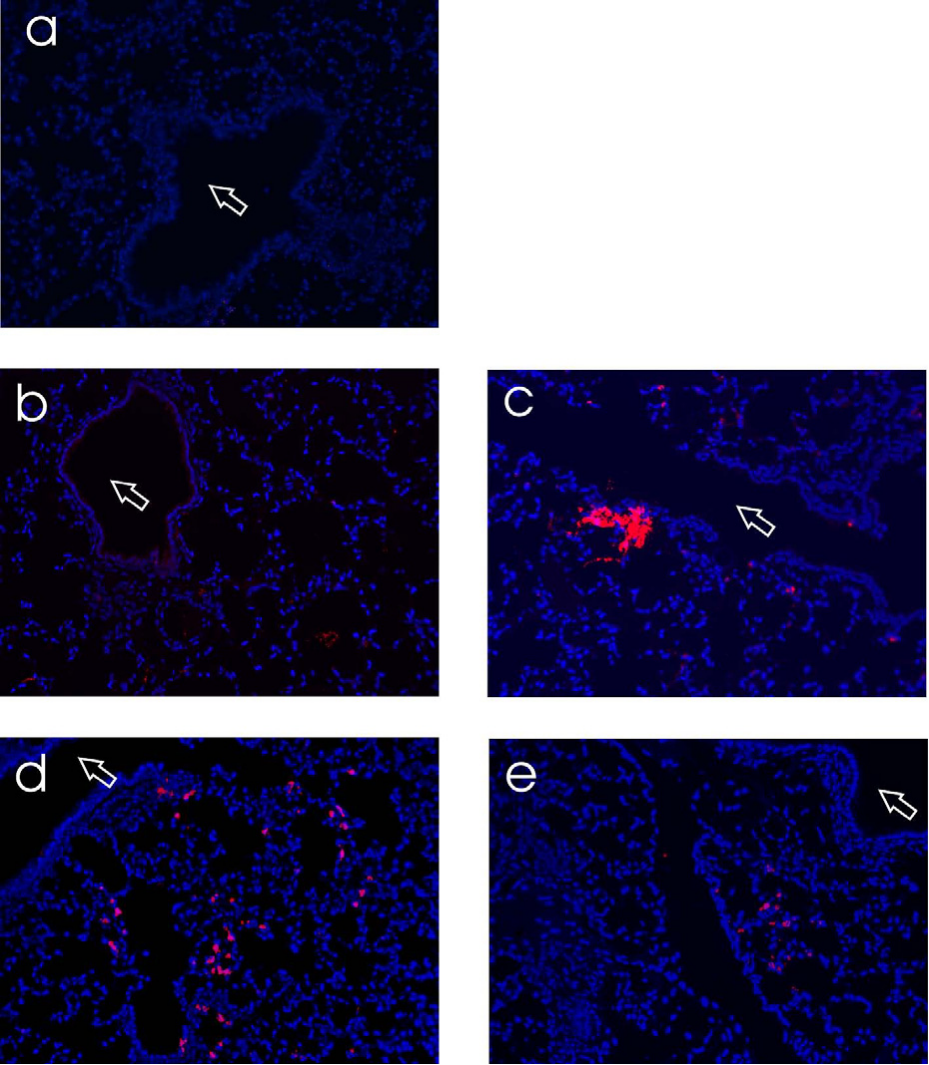
**103L, 106I and 103L+106I mutations enhance viral tropism in BALB/c lungs**. BALB/c mice were infected intransally with 10^5 ^pfu and lungs were collected 2 dpi. The lungs were inflated and fixed with 3.7% formaldehyde and lung sections were stained with anti-PR8 antibody. The bronchiolar regions are indicated by an arrow. **A**. Mock uninfected lungs **B**. rPR8-HK-NS-WT infected lungs **C**. rPR8-HK-NS-F103L infected lungs **D**. rPR8-HK-NS-M106I infected lungs **E**. rPR8-HK-NS-F103L + M106I infected lungs.

The recombinant PR8 viruses were used to assess the extent of infection and tropism in BALB/c mouse lungs infected with 10^5 ^pfu at 2 dpi by immunofluorescent staining. The lungs infected with the rPR8-HK-NS-wt showed a minimal pattern of detectable infection with scattered alveolar staining and no bronchiolar tissue infections (Figure 2b). The NS1 mutants showed an increased extent of alveolar tissue infection with larger foci in the lungs that was more pronounced in the single mutants rPR8-HK-NS-F103L and rPR8-HK-NS-M106I compared to rPR8-HK-NS-wt (Figure 2b, c, d, e), whereas no bronchiolar infections were observed for any of the mutants.

### Rate of viral protein synthesis

**Figure 3:**
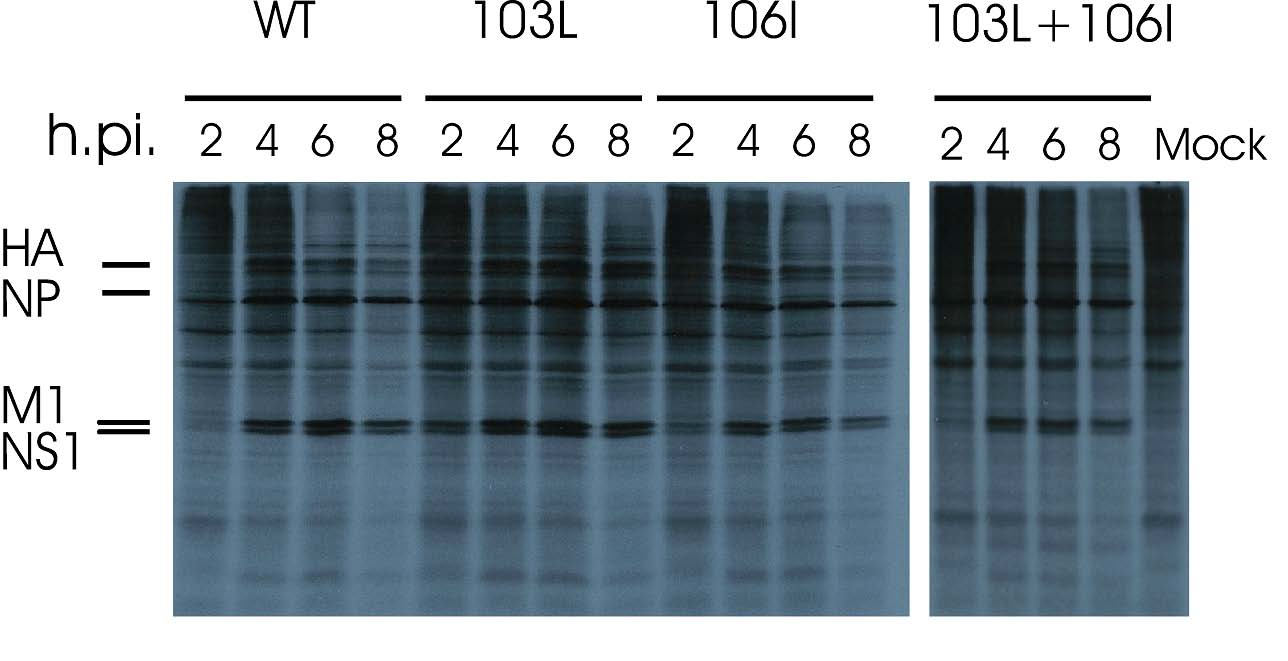
**Effect of F103L, M106I, and F103L + M106I NS1 mutations on the rate of viral protein synthesis in MDCK**. MDCK cells were infected with each of the rPR8 viruses at a MOI = 3 and pulsed for 1 hour with ^35^S at 2, 4, 6, and 8 hpi. Cell lysate was collected after pulse and used for SDS page and autoradiography. The experiment was performed twice and a representative SDS-page analysis is shown.

The mouse adapted NS1 mutations 103L and 106I map to binding sites for cellular translational (eIF4G-I and PABPI) and post-transcriptional factors (CPSF-30). To explore whether the enhanced growth and pathogenicity of the mutant viruses correlated with enhanced viral protein synthesis, the rate of viral protein synthesis was determined by ^35^S-met + cys labeling of infected MDCK cells. MDCK cells were infected with the different rPR8 viruses at MOI = 3 and cells were pulse-labeled with ^35^S met and ^35^S cys for 1 h at 2, 4, 6 and 8 hpi before SDS page and autoradiography. A representative gel for 1 of 2 independent experiments that demonstrated the same patterns is shown in Figure 3. The rate of viral protein synthesis was enhanced by the F103L mutation at all time points as demonstrated for the M1 and NS1 bands with the most dramatic increase shown early after infection (at 2 hpi). M106I also enhanced viral protein synthesis at 2 hpi but not at later time points (Figure 3). The double mutant produced similar or reduced levels of protein synthesis compared to rPR8-HK-NS-wt at all times (Figure 3). These data indicated that each of the F103L and M106I mutations resulted in more rapid gene expression seen at the level of protein synthesis, but that these mutations in combination were not as effective at enhancing protein synthesis.

### NS1 mouse adaptive mutations increase replication in untreated mouse cells and following IFN-β treatment

**Figure 4:**
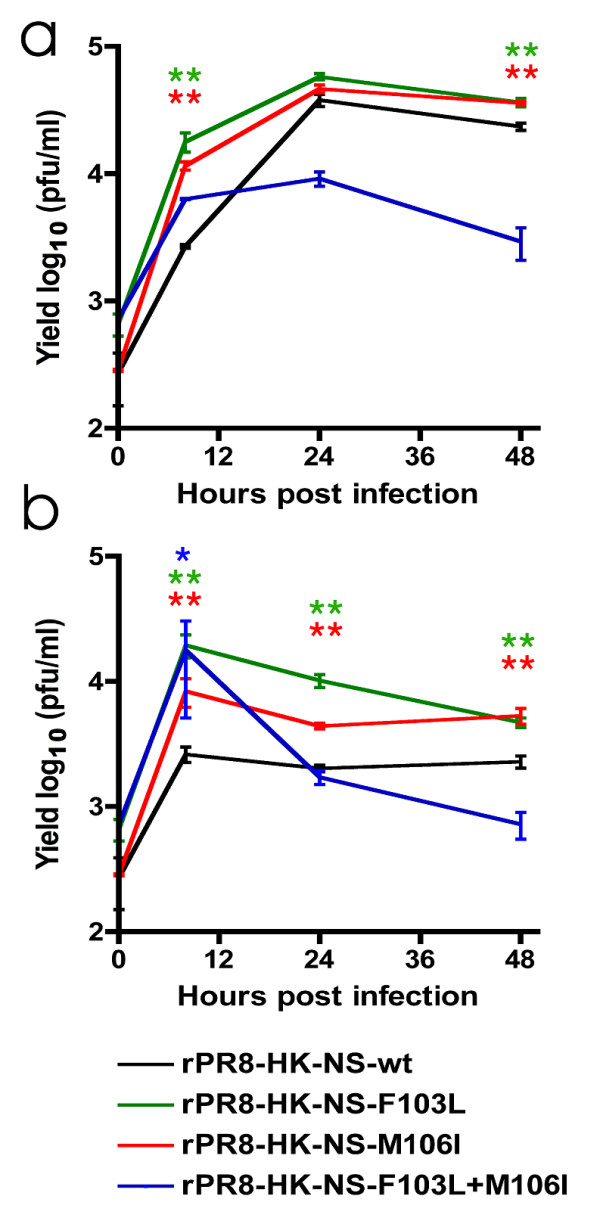
**Interferon sensitivity of the rPR8 viruses in mouse epithelial cell lines.** A. Growth curve of the rPR8 mutant viruses in M1 cells. Monolayers of M1 cells were infected with each of the rPR8 viruses at MOI = 1. Cell supernatants were collected at 0, 8, 24 and 48 hpi. Viral titer was assessed by plaque assay. B. Growth curve of the rPR8 mutant viruses in M1 cells in the presence of IFN-β. Monolayers of M1 cells were treated with 200U/ml of murine IFN-β. Twenty-four hours post treatment; monolayers were infected with each of the rPR8 viruses at MOI 1. Cell supernatants were collected at 0, 8, 24 and 48 hpi. Viral titer was assessed by plaque assay. Values are shown as average +/- standard deviation.

NS1 is involved in IFN antagonism as the NS1 deleted IAV A/PR/8/34 mutants is only capable of replicating in IFN unresponsive cells or mice such as Vero cells or in STAT-1 knock-out mice [43]. Therefore, to determine whether the difference in virulence and replication between the mutant viruses and the rPR8-HK-NS-wt was due to NS1 IFN antagonistic effect, the viral growth in mouse M1 cells was assessed in the presence and absence of IFN-β pre-treatment. Monolayers of M1 cells were either untreated or pre-treated with 200 U/ml of murine IFN-β for 24 hours. Twenty-four hours post treatment, cells were washed with PBS and infected in triplicate with the different rPR8 viruses at MOI = 1 in the presence of TPCK trypsin (1 μg/ml) and the cell supernatants were collected at 0, 8, 24 and 48 hpi. Without prior IFN-β treatment, the rPR8-HK-NS-wt reaches a peak of 3.8 × 10^4 ^pfu/ml at 24 hpi that was comparable to each of the single mutants at that time, while each of the mutations (103L and 106I) enhanced growth at the earliest (8 hpi) (by > 6 fold and >4 fold for the F103L and M106I mutants respectively) and at the latest time point (48 hpi) compared to rPR8-HK-NS-wt (P < 0.01) (Figure 4a). The double mutant grew better than the rPR8-HK-NS-wt at 8 hpi but to lower yields compared to each of the single mutants, and was reduced to lower than the rPR8-HK-NS-wt levels thereafter. Following IFN-β pretreatment, the rPR8-HK-NS-wt reached a peak of 3 × 10^3 ^pfu/ml at 8 hpi and all the viral mutants grew better than the rPR8-HK-NS-wt at this time (P < 0.01 for the single mutants and P < 0.05 for the double mutant) (>7 fold increase by F103L mutant). At later time points (24 and 48 hpi) only the single mutants 103L and 106I were able to enhance growth in the presence of IFN-β relative to rPR8-HK-NS-wt (P ≤ 0.01) (Figure 4b). Thus, following IFN-β treatment, the rPR8-HK-NS-F103L and rPR8-HK-NS-M106I mutants had demonstrated enhanced growth throughout infection relative to rPR8-HK-NS-wt, but enhanced growth was only seen at early times for the double mutant.

### 103L and 106I in H5N1 NS1 control virulence in BALB/c mice

**Figure 5:**
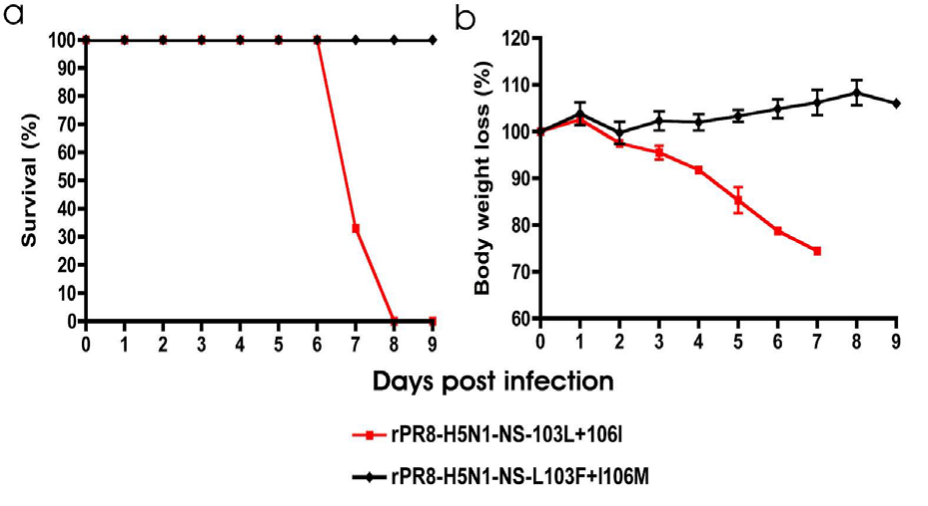
**Virulence and body weight loss are increased by 103L and 106I NS1 mutations in rPR8- H5N1-NS**. A. Groups of 3 BALB/c mice were infected intranasally with 10^4 ^pfu with the different rPR8 viruses. Survival was monitored for 9 dpi. B. The percent of body weight loss was calculated relative to body weights that were recorded daily throughout the whole course of the experiment.

To further assess the virulence associated with 103L and 106I sites, the H5N1 NS, that possess the L and I amino acids in the 103 and 106 sites respectively, was rescued onto a A/PR/8/34 backbone. The virulence of this virus was determined in BALB/c mice following intranasal infection with 10^4 ^pfu. The rPR8-H5N1-NS-103L+106I resulted in 100% mortality by 8 dpi indicating a LD_50 _of <10^3.5 ^(Figure 5a) that was associated with 26% body weight loss at the time of death (Figure 5b). We then mutated the 103L and 106L sites back to consensus and generated a PR8 recombinant possessing the H5N1-NS-L103F + I106M. The rPR8-H5N1-NS-L103F + I106M did not cause any mortality in mice, LD_50 _of >10^4.5 ^(Figure 5a); in addition, the infected mice only lost 4% of their body weight (Figure 5b). Therefore, the 103L and 106I are associated with severe disease and >10 fold increased virulence in the H5N1 NS1 gene.

### 103L and 106I in H9N2 NS1 control virulence in BALB/c mice

**Figure 6:**
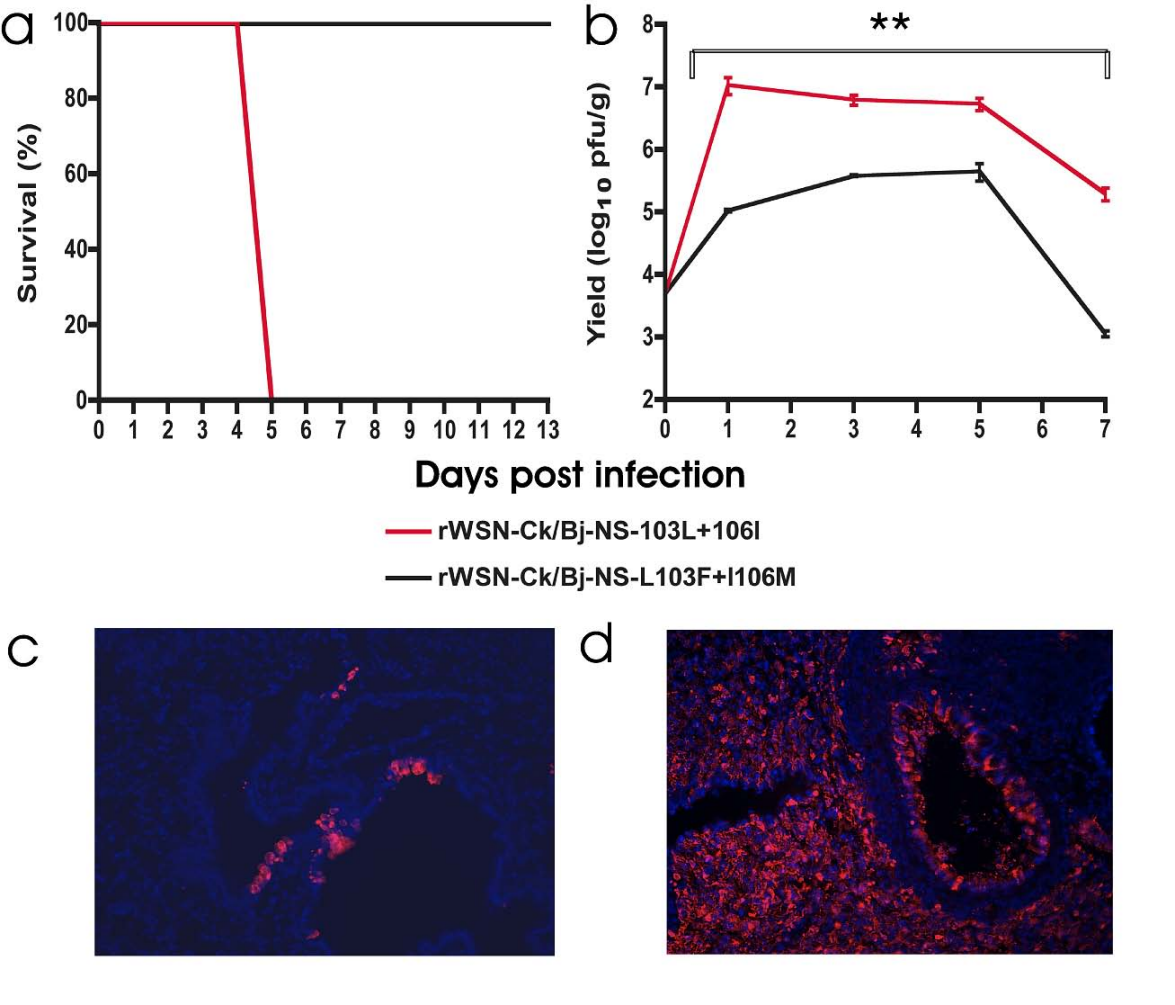
**Growth, virulence and viral tropism are increased by 103L and 106I NS1 mutations in rWSN-Ck/Bj-NS**. A. Groups of 5 BALB/c mice were infected intranasally with 10^6 ^pfu with the different rWSN viruses. Survival was monitored for 14 dpi. B. Groups of 5 BALB/c were infected intranasally with 5 × 10^3 ^pfu with the rWSN viruses. Lungs were collected at days 1, 3, 5 and 7 pi, sonicated and the viral titer was assessed by plaque assay. Values are shown as average of 2 replicates +/- standard error for each value. C. BALB/c mice were infected intransally with 10^5 ^pfu of rWSN-Ck/Bj-NS-L103F + I106M and lungs were collected 2 dpi. The lungs were inflated and fixed with 3.7% formaldehyde and lung sections were stained with anti-WSN antibody. The bronchiolar regions are indicated by an arrow. D. rWSN-Ck/Bj-NS-103L + 106I infected lungs. Statistical analysis was performed using the parameters of paired and two-tailed student's t-test with probability ≤ 0.01.

The properties of 103L and 106I mutations were also studied in the context of the Ck/Bj/95 (H9N2) NS gene, that possesses both 103L and 106I residues, on another mouse adapted backbone A/WSN/33 backbone. We tested this backbone because WSN NS1 inhibits IFN-β induction by a different mechanism that includes CPSF-30 binding in contrast to PR8 NS1 that does not bind CPSF-30 [44] (reviewed in [13]) and recent findings that multiple polymerase components act to block IFN induction [45,46] suggests that IFN antagonism is a function of multiple genes that may differ among strains. We then mutated the Ck/Bj/95 (H9N2) NS gene to possess L103F + I106M to generate the consensus sequence at these sites. The virulence and growth of the recombinant WSN virus having the unmodified H9N2-NS1 gene possessing 103L and 106I mutations was determined in BALB/c mice. The virus was highly virulent in BALB/c mice causing 100% mortality 5 dpi following intranasal infection with 10^6 ^pfu (Figure 6a). When both sites were mutated to L103F + I106M, the virus lost its ability to cause fatal infection at this dose in the mouse, as the virus did not cause any mortality throughout 14 dpi, thus the LD_50 _was reduced by >10 fold (Figure 6a).

The ability of the two rWSN viruses (rWSN-Ck/Bj-NS-103L + 106I and rWSN-Ck/Bj-NS-L103F + I106M) to grow in BALB/c mice was also determined by infecting groups of 8 mice intranasally at a lower infection dose of 5 × 10^3 ^pfu. The presence of the H9N2 NS1 103L + 106I residues increased growth in BALB/c lungs by 100 fold at 1 dpi and >10 fold at 3 and 5 dpi relative to the L103F + I106M mutant that was significantly different by paired t-test (P ≤ 0.01) (Figure 6b). The growth was also more persistent at 7 dpi with >2 logs increased lung titer due to the presence of F103L + M106I mutations (P < 0.05) (Figure 6b). Thus the Ck/Bj/95 NS1 F103L + M106I mutations conferred both increased replication and virulence in mouse lungs.

We also assessed the extent of lung infection using immunofluorescent staining of infected lung sections to show that the presence of L103F and I106M mutations in the Ck/Bj/95 H9N2-NS1 gene on the WSN backbone resulted in lung infection that was restricted to specific foci in the bronchiolar epithelium (Figure 6c). In contrast, the presence of 103L and 106I in the H9N2-NS1 dramatically enhanced viral spread and tropism in bronchiolar and alveolar tissues to encompass all of the alveoli except for regions around the bronchioles (Figure 6d). The extent of lung infection correlated with viral replication and virulence for both viruses in BALB/c mice. Thus we have identified two mutations in the NS1 gene that mediated increased replicative fitness and viral tropism in the mouse lung. Therefore the presence of Leu at position 103 and an Ile at position 106 are necessary and sufficient for a virus with a human (A/HK/1/68) on the A/PR/8/34 backbone or the avian (A/HK/156/97 and Ck/Bj/95) NS genes on either the A/PR/8/34 or A/WSN/1933 backbones respectively, to become virulent and enhance growth in BALB/c mice.

### Interferon induction by NS1 mutants in M1 cells

**Figure 7:**
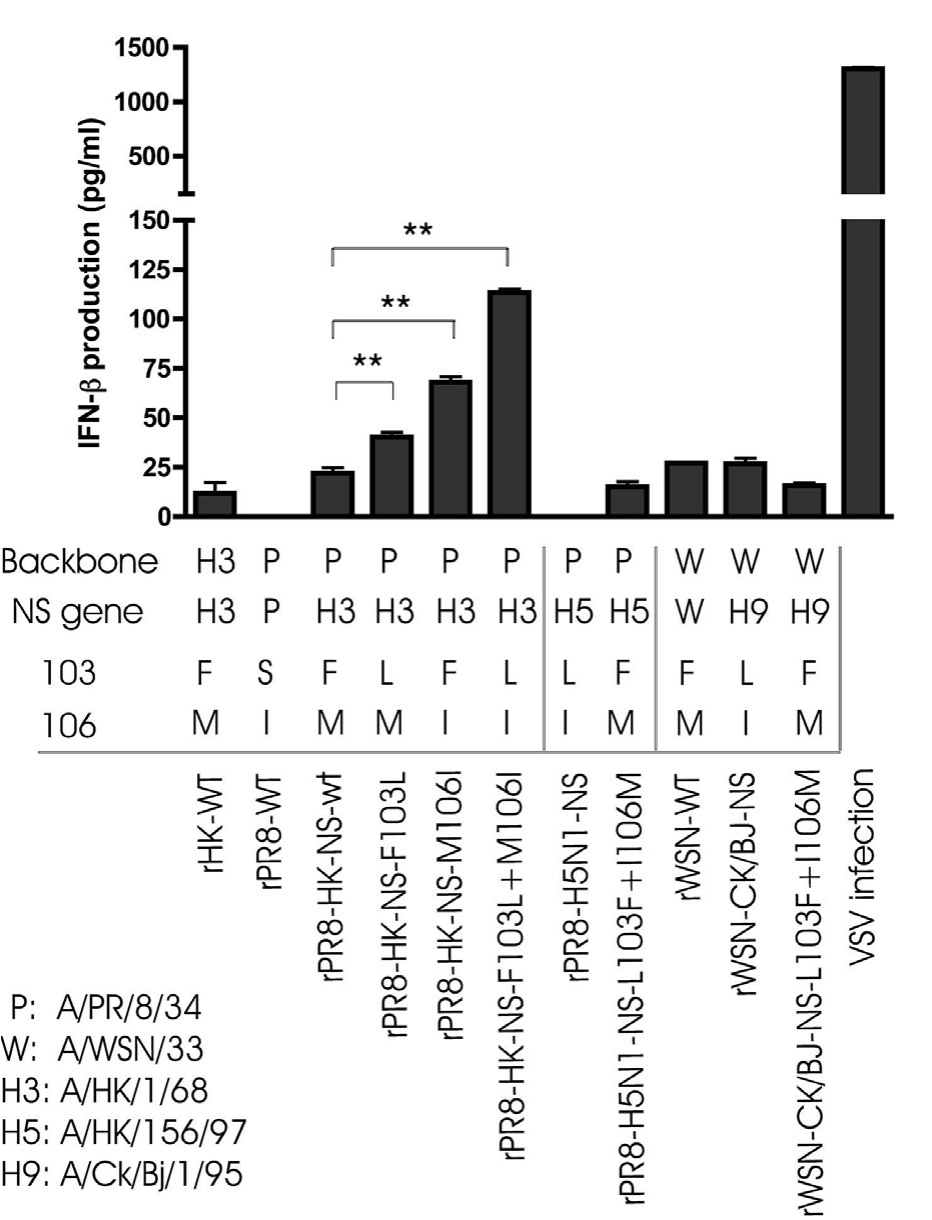
**Interferon production in M1 cells**. Monloayers of M1 cells were infected with the parental recombinant viruses rHK-wt, rPR8-wt and rWSN-wt and the different recombinant viruses, rPR8-HK-NS, rPR8-H5N1-NS and rWSN-Ck/Bj-NS viruses at MOI = 2. Twenty-four hours post infection, the supernatants were collected and the level of IFN-β production was assessed by ELISA. The IFN-β levels were compared to a positive control (VSV Δ51 infection). Values are shown as average +/- standard deviation.

As both the F103L and M106I mutations were associated with greater levels of growth than the double mutant at later times following IFN-β pretreatment, we assessed the amount of IFN-β production during infection, which could explain the differences in mutant gene functions among these mutants. To assess the roles of our NS mutants in controlling IFN-β production, monolayers of M1 cells were infected with each of the rPR8 viruses at MOI = 2. Twenty-four hours post infection, cell supernatants were collected and IFN-β production was assessed by ELISA. Infection with the HK-wt and the PR8-wt viruses resulted in low or undetectable levels of IFN-β respectively indicating that both NS1 (and possible roles of the NEP) proteins can suppress IFN-β induction on their respective genetic backbones. The rPR8-HK-NS-wt recombinant also induced low levels of IFN-β (22 pg/ml) whereas each of the recombinants rPR8-HK-NS-F103L or rPR8-HK-NS-M106I induced 2 and 3 fold more IFN-β respectively compared to rPR8-HK-NS-wt (40 and 68 pg/ml respectively) while the double mutations together had a cumulative effect on increasing IFN-β induction to 113 pg/ml (P < 0.001) (Figure 7). The increased IFN-β induction levels produced by the different mutants is consistent with the reduced viral growth at later times in M1 cells relative to rPR8-HK-NS-wt, when increasing amounts of IFN-β have accumulated to exert an antiviral effect (as shown by the effect of IFN-β pretreatment in Figure 4b). The extent of viral growth was thus inversely correlated with IFN-β induction where the rPR8-HK-NS-F103L virus grew to the highest yield in M1 cells in the presence and absence of IFN-β and induced the least amount of IFN-β, while the double mutant grew the least with and without IFN-β pre-treatment and induced the highest levels of IFN-β compared to the rest of the mutants. The increased IFN-β induction by the double mutant explains the reduced replication of this virus relative to the single mutants.

The levels of IFN-β induction in M1 cells following infection with the recombinants that possess the H5N1 NS and Ck/Bj/95 NS genes were also measured and showed minimal levels of IFN-β induction at 24 hpi. Interestingly, mutating the NS1 sites 103L and 106I to L103F and I106M in either of these NS1 genes resulted in viruses of low and comparable interferon induction indicating a role of other NS1 gene mutations in the regulation of IFN-β induction by these viruses. The effectiveness of the influenza NS1 mutants to inhibit IFN-β induction was shown in comparison with the VSV Δ 51mtuant that has a defect in its ability to block innate immunity and resulted in 1,306 pg/ml IFN-β. We can conclude that IFN-β induction is controlled by multiple genetic factors involving the nature of the NS1 gene as well as interactions with the genetic backbone.

## Discussion

Here we demonstrated that both the IAV NS1 F103L and M106I mutations were adaptive mutations that increased replicative abilities in cells of multiple species as well as virulence in the mouse lung. The observation that both the NS1 F103L and M106I mutations were under positive selection and were therefore adaptive mutations was evident by their increased prevalence in mouse adapted populations; where they were found in 3 of 6 [34] and 3 of 3 virus clones in independent mouse-adapted populations (Table 1). This indicated that these mutations conferred a selective advantage relative to the wild type NS1 genes possessing 103F and 106M. Although we observed a loss of ability to inhibit IFN-β induction on the PR8 backbone, this property was separable from an enhanced ability for protein synthesis and viral replication. Therefore, there appeared to be a counterbalance between the inhibitory effects of IFN-β induction and enhanced overall function seen at the level of replication and virulence for both of these mutations. In our experiments, the F103L and M106I mutations enhanced IFN-β induction only when transferred onto the foreign, A/PR/8/34, genetic background. This was also reported for the A/HK/156/97(H5N1) NS1 gene that also possessed these mutations and did not induce IFN-β on its native backbone but did when transferred onto the A/Udorn/1/72 (H3N2) backbone [32]; that was correlated with the loss of CPSF-30 binding and thus inhibition of host gene expression. The different abilities to inhibit IFN-β induction among viruses, that possessed the same combinations of mutations, 103L and 106I versus 103F and 106M, but that differed in their NS1 genes (A/HK/1/68, A/HK/156/97 (H5N1) or Ck/Bj/95 (H9N2)) as well as virus backbones (PR/8/34 and WSN/33), indicates that inhibition of IFN-β induction is not only controlled by the NS1 gene but also by epistatic interactions with the other virus gene products. It has been reported that the A/HK/156/97(H5N1) was able to suppress IFN-β induction in the context of its NS1 gene, possessing F103L and M106I mutations, due to properties of its NP and PA genes [47]. This is consistent with the recently described roles for PA, PB1 and PB2 genes in blocking IFN induction by binding to the mitochondrial antiviral signaling protein (MAVS) [45,46] but suggest that a functional interaction with NS1 protein is also involved in these (or other undefined) processes which are downstream from the RIG-I signal that is inhibited by NS1 [48]. Thus the F103L and M106I mutations were not associated with increased IFN-β induction either in A/HK/156/97(H5N1) virus [47] or for the MA20c and MA51 mouse-adapted viruses in which these mutations were selected, indicating that these mutations were selected in a genetic context that maintains suppression of IFN-β induction. Increased IFN-β induction was also suppressed for the H9N2 NS gene on the WSN backbone, and the H5N1 NS gene on the PR8 backbone where it was demonstrated that in the absence of increased IFN-β induction the F103L and M106I mutations mediated a large increase in extent of lung infection and virulence. We have therefore demonstrated that both F103L and M106I mutations were multifunctional, including loss of ability to suppress IFN-β in some contexts but also including gain-of-function that increased replicative abilities and virulence. Multifunctional adaptive mutations have been previously seen in the HA gene for individual adaptive mutations that affect both pH of fusion and receptor binding [8] suggesting that strongly adaptive mutations affect more than one function.

### Mechanisms of action of NS1 103L and 106I mutations

The NS1 mouse adaptive mutations demonstrated an increased ability to antagonize IFN-β activity observed by the enhanced ability to grow in mouse epithelial cells following IFN-β pre-treatment when compared to rPR8-HK-NS-wt. Increased growth of these mutants was also greater at early times in MDCK and mouse M1 cells consistent with greater replication at early times before IFN-β had been induced in the infected cell cultures. Although CPSF binding has been shown to result in inhibition of IFN and other host gene expression [18], A/PR/8/34 (H1N1) viruses have been shown to have lost CPSF binding due to mutations at positions 103 and 106 (S + I) and yet maintains the ability to block IFN induction by preventing pre-transcriptional activation of IRF-3, NFκB and c-Jun/ATF-2 as reviewed [13]. Given that the 103 and 106 amino acids also map to regions of interaction between NS1 and cellular translational initiation factors eIF4GI and PABPI, leads to the hypothesis of increased NS1-mediated initiation complex formation on IAV mRNA for enhanced viral gene expression due to mutations F103L and M106I. It is also possible that these mutations affect other functions of NS1 that are mediated by interaction with other viral or host proteins. Several studies have identified multiple host factors that interact with NS1 protein [13], including the recent identification of nine PR8 viral proteins and 20 host proteins that bind NS1 protein, all of which may be modified in their interaction due to adaptive mutations [14]. Future studies are needed to assess the role of the adaptive HK-NS1 mutations on interactions with other viral factors.

### F103L and M106I Mutations enhance protein synthesis

The increased protein synthesis of the F103L NS1 mutant was clearly seen for the M1 and NS1 proteins at all times in MDCK cells whereas the M106I mutation increased protein synthesis at 2 hpi but not at later times, and where the double mutant was not significantly enhanced relative to rPR8-HK-NS-wt (Figure 3). This may have been due to the increased induction of IFN-β as seen for mouse cells infected with these mutants. Thus there was an enhancement of protein synthesis due to each mutation alone but that this was being suppressed by the increased amounts of IFN-β that were simultaneously expressed for F103L and M106I in combination, thus indicating the critical role of NS1 in inhibition of IFN-β induction.

### The F103L and M106I mutations increased virulence of human and avian NS1 genes

Both HK-NS1 F103L and M106I mutations enhanced the level and duration of replication resulting in increased virulence in the mouse lung where viral titers were 10^4 ^to 10^5 ^fold higher at 5 dpi in infected lungs, in contrast to rPR8-HK-NS-wt, which had viral titers below the level of detection. Immunofluorescent staining showed that each mutation in the HK-NS1 gene enhanced the ability to infect clusters of cells in the alveoli of infected mice. The extent of infection as monitored by virus yield suggested that more cells were infected in the lung for all the mutant viruses but that both mutations in combination were not more effective in the PR8 backbone than the F103L mutation alone, presumably because of the increased amount of induced IFN-β by this mutant. We also demonstrated that these mutations were critical for the enhanced virulence of the HK/156/97 and Ck/Bj/95 NS1 genes such that reversion to L103F and I106M resulted in an IAV that was very restricted in its ability to infect lung tissues. These mutations were not associated with increased IFN-β induction in M1 cells when present on the Ck/Bj/95 NS1 gene on the WSN background, or on the A/HK/156/97 NS gene on the A/PR/8/34 backbone indicating that the ability to block IFN-β induction was maintained by these avian NS genes on the WSN or PR8 backgrounds. The NS1 F103L and M106I mutations mediated a large expansion of cell tropism for rWSN-Ck/Bj-NS-103L + 106I where the virus infected a much larger proportion of the lung. This effect is consistent with a greater ability to resist inhibition by IFN-β and paralleled the large effect of the PR8 NS1 gene on expanding tissue tropism that was observable in PR8 infected STAT1 knockout mice when compared to virus that had the NS1 gene deleted in the PR8 NS1 null mutant [43].

## Conclusion

In summary, we have identified two multifunctional mutations in the NS1 gene that control virulence. The presence of a Leu at position 103 and Ile at position 106 were selected in mice and independently in avian IAV lineages and are required for an increase in virulence and growth in both human A/HK/1/68 and avian (Ck/Bj/1/95 and A/HK/156/97) NS genes as shown on multiple genetic backgrounds (A/WSN/1/33 as well as A/PR/8/34). Therefore, we conclude that the NS1 protein of IAV is a virulence determinant that in addition to controlling IFN-β induction mediates gain-of-function effects on both protein synthesis and viral replication due to the presence of 103L and 106I that increase the extent of lung tropism and infection.

## Competing interests

The authors declare that they have no competing interests.

## Authors' contributions

SKD participated in the conception and design of the study, data acquisition, analysis and interpretation of data as well as drafting the manuscript. SW participated in the acquisition, analysis and interpretation of data. JP participated in the acquisition, analysis and interpretation of data. NEF participated in the acquisition, analysis and interpretation of data. LK participated in the acquisition, analysis and interpretation of data. YL participated in the acquisition, analysis and interpretation of data. EGB contributed to the study's conception and design, data acquisition, analysis and interpretation of data, drafting the manuscript and general supervision of the research group. All the authors have read and approved the final manuscript

## Authors' information

EGB is a professor in the University of Ottawa, Department Of Biochemistry, Microbiology and Immunology. SKD, SW and NEF are PhD students at the University of Ottawa, Department Of Biochemistry, Microbiology and Immunology, Ottawa, Ontario, Canada. JP and LK are post-doctoral fellows at the University of Ottawa, Department Of Biochemistry, Microbiology and Immunology, Ottawa, Ontario, Canada. YL is a MSc. graduate from the University of Ottawa, Department Of Biochemistry, Microbiology and Immunology and currently employed with Health Canada, Ottawa, Ontario, Canada.
